# Mitochondrial Cardiolipin-Targeted Tetrapeptide, SS-31, Exerts Neuroprotective Effects Within In Vitro and In Vivo Models of Spinal Cord Injury

**DOI:** 10.3390/ijms26073327

**Published:** 2025-04-02

**Authors:** Baylen Ravenscraft, Do-Hun Lee, Heqiao Dai, Abbie Lea Watson, Gabriela Inés Aparicio, Xianlin Han, Ling-Xiao Deng, Nai-Kui Liu

**Affiliations:** 1 Indiana Spinal Cord and Brain Injury Research Group, Stark Neurosciences Research Institute, Department of Neurological Surgery, Indiana University School of Medicine, Indianapolis, IN 46202, USAdohun.lee@bcm.edu (D.-H.L.);; 2 Department of Neuroscience, Baylor College of Medicine, Houston, TX 77030, USA; 3 Neurorestoration Center, Department of Neurosurgery, University of Kentucky College of Medicine, Lexington, KY 40536, USA; 4 Department of Medicine, University of Texas Health Science Center at San Antonio, San Antonio, TX 78229, USA; hanx@uthscsa.edu

**Keywords:** spinal cord injury, cardiolipin, SS-31, neuroprotection, mitochondrial function, lipid

## Abstract

Spinal cord injury (SCI) affects millions globally, leading to severe motor and sensory deficits with no effective clinical treatment. Cardiolipin (CL), a mitochondria-specific phospholipid, plays a critical role in bioenergetics and apoptosis. Emerging evidence suggests that CL alterations contribute to secondary SCI pathology, but their precise role and underlying mechanisms remain fully understudied. In this study, we investigated the protective effects of SS-31 on CL alteration, neuronal death, tissue damage, and behavioral recovery after SCI using both in vitro and in vivo models, lipidomics analysis, histological evaluation, and behavioral assessments. In vitro investigations used primary spinal cord neuron cultures, challenged with either rotenone or glutamatergic excitotoxicity, with protective capabilities measured via cell death assays and neurite morphological analysis. In vivo investigations used female adult C57Bl/6 mice, challenged with a contusive SCI. The results showed that SS-31 reduced rotenone- and glutamate-induced mitochondrial dysfunction and neuronal death in a dose-dependent manner in vitro. Additionally, SS-31 attenuated rotenone- and glutamate-induced neurite degeneration in vitro. Lipidomics analysis revealed a reduction in CL at 24 h post-SCI in adult mice, which was attenuated by SS-31 in a dose-dependent manner. Consistent with this effect, SS-31 improved behavioral recovery after SCI in adult mice, although it had no significant effect on tissue damage. These findings suggest that CL alteration may play a key role in the pathogenesis of SCI, at least in the C57BL/6 mouse, and as such could be an attractive therapeutic target for ameliorating secondary SCI.

## 1. Introduction

Spinal cord injury (SCI) is a major global health issue that affected an estimated 15.4 million people globally, contributing to over 4.5 million years of life lived with disability, in 2021, with diverse effects, including sensory and motor loss below the level of injury, as well as devastating consequences on the psyche of the patients, and severe financial burden for the cost of healthcare [1,2,3]. Despite extensive research, there is currently no effective treatment available for clinical cases of SCI [4].

The pathophysiology of SCI involves both primary and secondary injury mechanisms. The primary injury results from the initial mechanical trauma, while the secondary injury involves a cascade of biochemical and cellular events, including inflammation, oxidative stress, and mitochondrial dysfunction, which exacerbate tissue damage and functional deficits [5,6,7,8]. Among these, alterations in phospholipid metabolism, particularly the degradation of membrane phospholipids, play a critical role in the progression of secondary injury [5,6,7,9,10]. Phospholipids are fundamental components of cell membranes and, in neural cells, they are essential for maintaining membrane integrity, ion permeability, and protein functionality [11,12,13]. One of the first pathophysiological events occurring following SCI is the release of free fatty acids due to phospholipid degradation by the activation of phospholipase A_2_ (PLA_2_) [6], which leads to bioenergetic deficits and oxidative damage. This lipid dysregulation not only exacerbates mitochondrial dysfunction but also alters inflammatory responses, contributing to secondary injury cascades [9,10]. Although alteration of phospholipid metabolism has long been associated with SCI, its specific role in mediating damage and signaling cascades remains largely unknown [6,14,15].

Cardiolipin (CL) is a unique type of diphosphatidyl-glycerol phospholipid that has a doubly anionic (-2) head group, four acyl chains, and an overall cone-like structure [13]. Reminiscent of the endosymbiotic theory, it is present in bacteria, mitochondria, and related organelles (chloroplasts and mitoplasts), with an asymmetric distribution of around 90% within the inner leaflet of the inner mitochondrial membrane (IMM) [15,16]. Critical roles have been attributed to CL across healthy and pathological states, with particular emphasis on bioenergetics and cell stress responses [17,18]. The alteration of CL, such as by peroxidation or hydrolyzation, has been identified as having roles in diverse pathologies, including neurotrauma [19,20,21,22,23]. Although increasing evidence suggests that CL alteration may contribute to secondary SCI, its precise role in SCI remains to be delineated. Our previous report demonstrated that administration of XJB-5-131, a novel mitochondria-targeted electron and reactive oxygen species scavenger, effectively attenuated CL alterations, reduced cell death and tissue damage, and ameliorated motor deficits after SCI in adult rats. However, this does not completely exclude the possibility that XJB-5-131’s protective effects may also involve the mitigation of other mitochondrial oxidative factors. SS-31 (D-Arg-dimethylTyr-Lys-Phe-NH2, also known as Bendavia or Elamipretide) is a direct CL-protective compound [24,25], which binds selectively to CL, and protects CL by preventing it from converting cyto *c* into a peroxidase, while protecting its electron carrying function [25,26,27]. Although several studies have investigated SS-31 treatment in animal models of SCI [22,28,29,30,31], their results and reported effects have been inconsistent. Notably, none of these studies examined changes in CL following SS-31 treatment. This gap in knowledge highlights the need for further investigation into the role of CL in SCI and the therapeutic potential of SS-31. Here, we report that lipidomic analysis revealed a reduction in CL at 24 h after SCI in adult mice, and this change was attenuated by SS-31 in a dose-dependent manner. Furthermore, both in vitro and in vivo models of SCI demonstrated the neuroprotective effects of SS-31, including reduced mitochondrial dysfunction and neuronal death, and improved functional recovery after SCI, although it had no effect on tissue damage in the mouse SCI model.

## 2. Results

### 2.1. SS-31 Protected Spinal Cord Neurons from In Vitro Cell Death Induced by Rotenone or Glutamatergic Excitotoxicity

**Figure 1:**
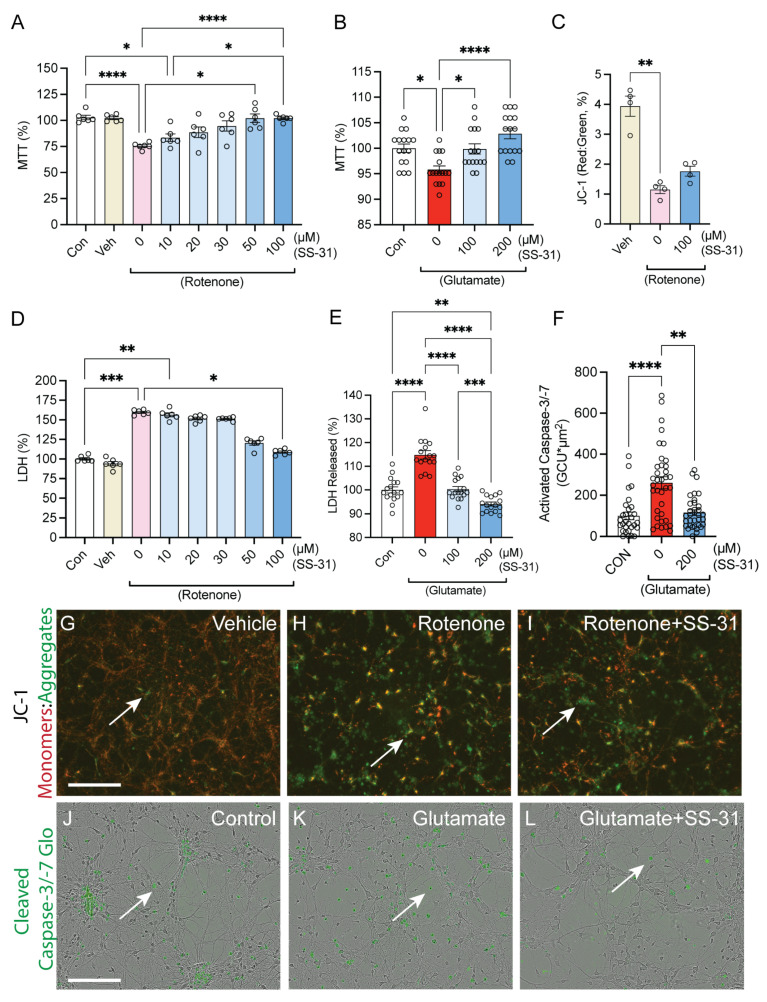
**Effects of SS-31 on rotenone- and glutamate- induced cell death in vitro.** (**A**–**E**) The spinal neuronal cultures were exposed to rotenone (125 nM, (**A**,**D**) pink) or glutamate (100 μM, (**B**,**E**) red), either in the absence or presence of the designated concentrations of SS-31, for 24 h (blue). SS-31 was added 30 min before rotenone or concomitantly with glutamate administration. SS-31 dose-dependently reversed the reduction in spinal cord neuronal cell viability caused by rotenone or glutamate (**A**,**B**), as measured by the MTT assay, and reduced spinal cord neuronal death induced by these agents (**D**,**E**), as indicated by LDH release. (**C**,**F**) SS-31 also reversed rotenone-induced mitochondrial dysfunction, as measured by the JC-1 assay (**C**,**G**–**I**), and glutamate-induced apoptosis, evidenced by the increased number of activated caspase-3/7 cells (**F**,**J**–**L**). *: *p* < 0.05, **: *p* < 0.01, ***: *p* < 0.001, ****: *p* < 0.0001. (**A**,**E**) Brown–Forsythe and Welch ANOVA and Dunnett’s T3 Multiple Comparison. (**B**–**D**,**F**) Kruskal–Wallis ANOVA and Dunn’s Multiple Comparison Test). Data represent the mean ± s.e.m. (**G**–**I**) Representative images show mitochondrial membrane potential (MMP, Δψm) indicated by the red fluorescence (J-aggregates, 590 nm) and green fluorescence (J-monomers, 525 nm), collected at 24 h following treatment. A decrease in the ratio of red to green fluorescence indicates a reduction in mitochondrial membrane potential (MMP, Δψm), which is also a marker of mitochondrial dysfunction (arrows). Bar = 200 μm. (**J**–**L**) Representative images show apoptotic cells with activated caspase-3/7, indicated by bright green nuclei (arrows), collected at 4 h following treatment. Bar = 200 μm.

To assess whether CL alteration contributes to mitochondrial dysfunction and neuronal death after SCI, we examined the effects of SS-31, a CL-protective compound, on glutamate- and rotenone-induced toxicity in primary spinal cord neurons (Figure 1). Rotenone has been shown to induce mitochondrial CL alteration [20,32]. In vitro viability was assessed with the MTT assay. Results showed that rotenone induced a 27% loss of viability, which was returned to baseline levels in a dose-dependent manner after administration of SS-31 (Figure 1A, Brown–Forsythe and Welch’s ANOVA tests’ *p*-values < 0.0001, *n* = 6/group). The glutamatergic excitotoxic injury of 100 μM induced a 4.2% decrease of the MTT assay, which was reversed with coadministration of SS-31 (Figure 1B, Kruskal–Wallis ANOVA test *p*-value < 0.0001, *n* = 16/group).

The JC-1 assay was used to measure SS-31-mediated protection of mitochondrial membrane potential (MMP) following rotenone administration. MMP is an important parameter of mitochondrial function [20,33,34,35]. JC-1 is a lipophilic, cationic dye that can selectively enter mitochondria and reversibly change color from green (525 nm) to red (590 nm) as the membrane potential increases. With normal mitochondrial function, MMP level is high and the red fluorescence (J-aggregate) is predominant. However, mitochondrial injury reduces MMP leading to an increase in green fluorescence [33]. Shown in Figure 1C, the control group presented about a 4:1 ratio of cell-confluence-normalized fluorescence of red monomers to green aggregates, and that was significantly reduced to about a 1:1 ratio when assessed 24 h following the administration of the rotenone injury, which was then attenuated to the insignificant ratio of about 1.75 when SS-31 was added 30 min following the administration of rotenone (Figure 1C, Kruskal–Wallis ANOVA test *p*-value < 0.0005, *n* = 4/group). Representative images of these cultures are shown in Figure 1G–I, with white arrows highlighting green polymers.

The LDH assay was used to measure levels of cell death by assessing the extracellular levels of the intracellular lactate dehydrogenase (LDH) enzyme, indicating relative levels of dying cells with permeable plasma membranes. Compared to the control cells, Rotenone treatment increased the levels of extracellular LDH detected by about 60% after 24 h, and that was attenuated, in a concentration-dependent manner with SS-31, to 100 μM. which resulted in a 9% increase compared to control wells (Figure 1D, Kruskal–Wallis ANOVA test *p*-value < 0.0001, *n* = 6/group). When the glutamatergic excitotoxicity model was used, there was a 15% increase in LDH detected at 24 h post-injury, and that was completely prevented with SS-31 (Figure 1E, Brown–Forsythe and Welch’s ANOVA tests’ *p*-values < 0.0001, *n* = 16/group).

To determine whether the protective effects of SS-31 were due to the prevention of apoptosis, the Cleaved Caspase-3/-7 glo kit (CC37) was used to detect activated caspases 3 and 7, which are downstream of the mitochondrial steps of apoptosis [36]. Compared to control cells, exogenous glutamate induced a 2.6-fold increase of CC37 detection 4 h following glutamate administration, and that was significantly reduced to a 0.16-fold increase when SS-31 was added at the same time as the glutamate (Figure 1F, Kruskal–Wallis ANOVA test *p*-value < 0.0001, *n* = 33/group). Representative images of these cultures are shown in Figure 1J–L, with white arrows highlighting the green fluorescence of activated caspases. These findings suggest that CL alteration could induce neuronal death after SCI, potentially through apoptosis.

### 2.2. SS-31 Protected Spinal Cord Neurons from In Vitro Neurite Degeneration Induced by Glutamatergic Excitotoxicity

**Figure 2:**
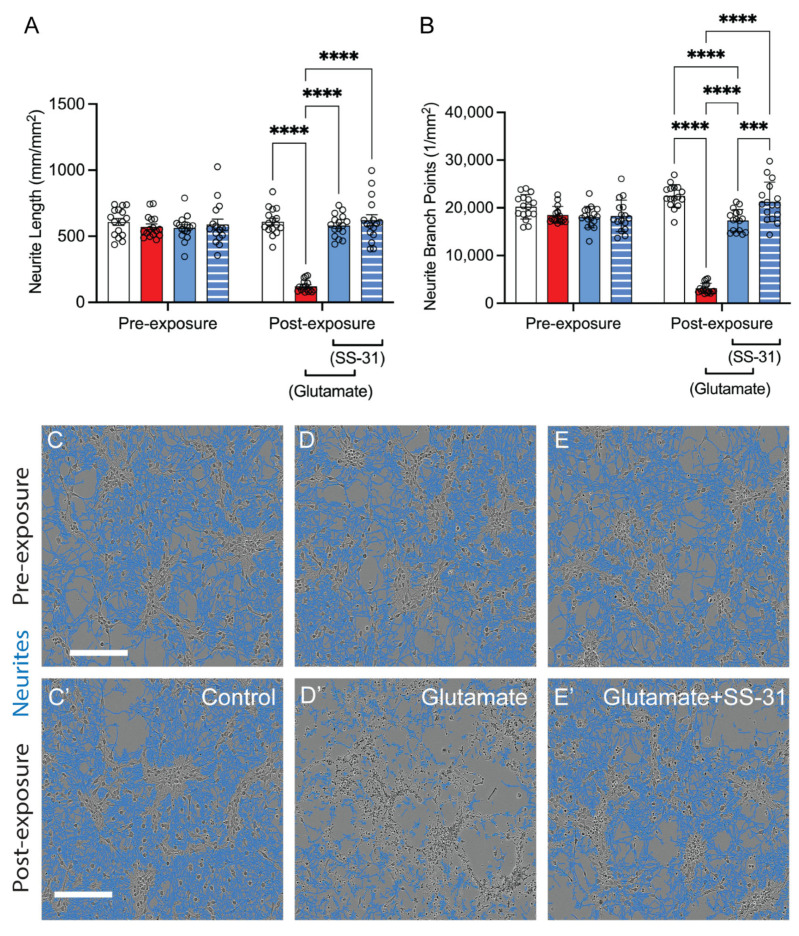
**Effects of SS-31 on glutamate-induced neurite degeneration in vitro.** (**A**–**E’**) The spinal neuronal cultures were exposed to glutamate (100 μM) either in the absence or presence of SS-31 (100 μM) for 24 h. SS-31 was added at the same time as glutamate administration. Pre-exposure refers to neurons before glutamate exposure and Post-exposure refers to neurons after glutamate treatment with or without SS-31. Bar graphs show that SS-31 reversed the glutamate-induced reduction in neurite length (**A**) and the decrease in neurite branches (**B**). ***: *p* < 0.001, ****: *p* < 0.0001 (Mixed-effects ANOVA, Tukey’s multiple comparisons test). Data represent the mean ± s.e.m. (**C**–**E’**) Representative images show cultured spinal cord neurons and their neurites (blue), from immediately prior to treatment (**C**–**E**) and 24 h following treatment (**C’**–**E’**). Panels (**C’**–**E’**) represent post-exposure conditions corresponding to their untreated pre-exposure counterparts (**C**–**E**). Bar = 200 μm.

To determine whether SS-31 protects the morphology of neurites, an Incucyte was used to image the cultures immediately prior to injury treatment and 24 h post-injury. Neurotrack software (v.2015A1.1) was used to quantify the average length and branch points of neurites. No significant difference was detected across the groups in pre-exposure (Figure 2A–E). However, at 24 h post-exposure, there was an 80% loss of neurite length and an 86% loss of neurite branch points measured in the glutamate-treated cultures (Figure 2A,B,D). That loss was strongly attenuated with the coadministration of 100 μM of SS-31, which resulted in a 4.82% loss of neurite length and a 22.8% loss of neurite branch points compared to the post-exposure control group (Figure 2A,B,E’). (Figure 2A,B, mixed-effects ANOVA test time, column-factor, and time column factor, ****: *p* < 0.0001, *n* = 16/group). Representative images with blue masking highlighting detected neurites are provided in Figure 2C–2E’.

### 2.3. SS-31 Attenuated Cardiolipin Alterations in the Injured Spinal Cord After In Vivo SCI

**Figure 3:**
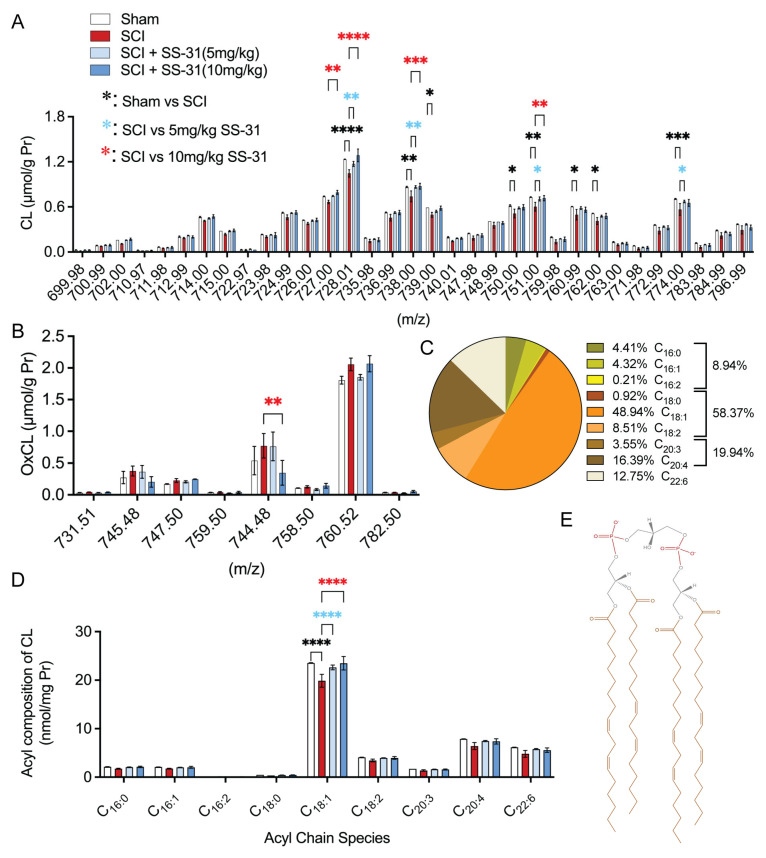
**Cardiolipin (CL) alteration after spinal cord injury (SCI) in adult mice.** (**A**) Quantification of CL molecular species at 24 h after SCI revealed significant reductions in many species, which were often dose-dependently reversed by SS-31. (**B**) Species of oxidized CL at 24 h after SCI. (**C**) The percentage graph shows the acyl composition of CL species in different grouping from Sham treatment group. (**D**) Quantification of acyl composition in CL species at 24 h after SCI. *: *p* < 0.05, **: *p* < 0.01, ***: *p* < 0.001, ****: *p* < 0.0001 (Two-way ANOVA, Tukey’s multiple comparisons test, *n* = 3 mice/group). Data represent the mean ± s.e.m. (**E**) Schematic diagram of a tetra-octadecadienoic (18:1)_4_ CL with orange-colored (18:1) acyl chains that have different unsaturation configurations (sn1(1′)-4*E*, sn2(1′)-9*Z*, sn1(3′)-13*Z*, sn2(3′)-17*Z*), red-colored phosphate headgroups, and black glycine linker groups.

Lipidomic analysis in our previous publication [20] showed a significant reduction in CL molecular species with *m*/*z* 714.00, 727.00, 728.01, 736.99, 738.00, 739.00, 750.00, 751.00, 759.98, 760.99, 762.00, 763.00, 774.00, and 796.99 at 3 and 24 h post-SCI in adult rats. To further investigate the role of CL alterations in SCI, we first determined the effective dose of SS-31 on CL alteration in a contusive mouse SCI model. Our lipidomic analysis revealed that SS-31 attenuated SCI-induced CL loss in a dose-dependent manner (Figure 3) at 24 h post-injury. Lipidomic analysis was conducted on 1 cm epicenter-centered segments of the mouse spinal cord, harvested and flash frozen at 24 h post-injury. The type of lipidomics employed in this study (electrospray ionization mass spectrometry) was limited to identifying groups of CL with the same mass-to-charge ratios (*m*/*z*), yet across the detection range (699.98–796.99 *m*/*z*) there was a general pattern of SCI-induced loss of CL, and of SS-31 administration reversing that effect, often back to baseline levels, and in many cases with a statistically significant dose-dependent effect (727.00, 728.01, 738.00, 751.00 *m*/*z*) (Figure 3A). It has been reported that CL is the only phospholipid in mitochondria that undergoes early oxidation after injury [37] or during apoptosis [38]. This oxidation is facilitated by the CL-specific peroxidase activity of cytochrome c bound to CL [38]. Additionally, oxidized CL (OxCL) triggers the release of pro-apoptotic factors, initiating the apoptosis cascade [38]. Our results further showed that the only statistically significant effect was with the 10 mg/kg treatment, reducing OxCL at 744.48 (*m*/*z*) with a similar, albeit statistically insignificant, result at 745.48 (*m*/*z*). Interestingly, at 760.52 (*m*/*z*), the 5 mg/kg group demonstrated protection towards baseline levels while the 10 mg/kg did not, although no statistical significance was found (Figure 3B). The percentages of acyl composition detected in the control group are presented as a pie chart in Figure 3C, with 48.94% belonging to mono-unsaturated octadecenoic acid (18:1) and the next two highest concentrations detected the longer poly-unsaturated fatty acids, arachidonic acid (20:4) at 16.39%, and docosahexaenoic acid (22:6) at 12.75%. In this study, lipidomic analysis revealed that the profile of molecular species and acyl composition of CL in mice (Figure 3C,D) closely resembles that observed in rats in our previous publication [20]. In uninjured spinal cord tissue, approximately 50% of CL in both species was composed of 18:1 fatty acids, while polyunsaturated fatty acids accounted for about 40% in both rats and mice. When comparing these acyl chain measurements across treatment groups, SS-31 was found to provide statistically significant protection of the predominant (18:1) acyl chain (Figure 3D). An illustration of an example tetra-oleoyl (18:1)_4_ CL (TOCL) is shown in Figure 3E.

### 2.4. SS-31 Improved Functional Locomotor Recovery After SCI

**Figure 4:**
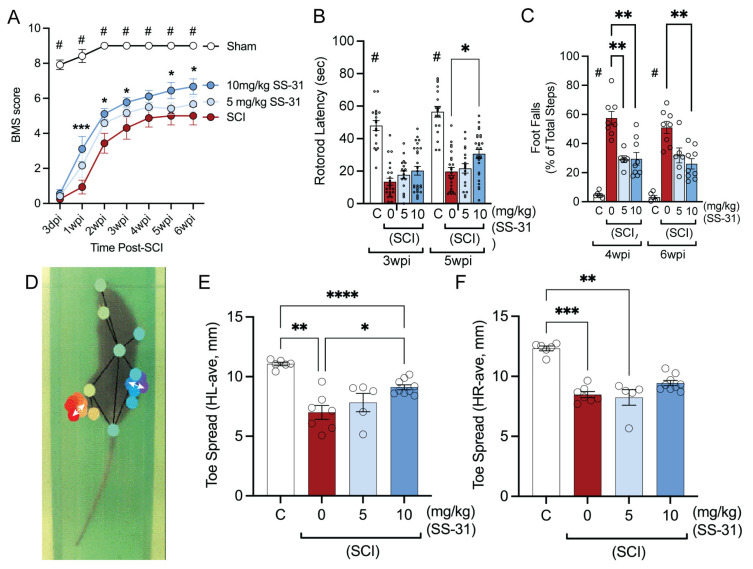
**Effect of SS-31 on behavioral outcomes after spinal cord injury (SCI) in adult mice.** (**A**) Administration of SS-31 improved BMS locomotor scores up to 6 weeks post-SCI in adult mice in a dose-dependent manner. #: *p* < 0.0001 vs. all of other groups; *: *p* < 0.05, ***: *p* < 0.001 vs. vehicle-treated groups (Two-way ANOVA, Tukey’s multiple comparisons test, *n* = 6–9 mice/group). (**B**) SS-31 significantly improved rotarod performances at 5 weeks post-injury. * *p* < 0.05 (Mixed-effects ANOVA, Tukey’s multiple comparisons test, *n* = 18–27/group). (**C**) SS-31 significantly reduced foot falls at 4 and 6 weeks post-injury. **: *p* < 0.01 (Mixed-effects ANOVA, Tukey’s multiple comparisons test, *n* = 6–9 mice/group). (**D**) Image of TreadScan perspective with DeepLabCut labels and white arrows showing the distance of the hindlimb toes pre-ad. (**E**,**F**) Quantifications of average hindlimb toes spread of the left (**E**) and right (**F**) hindlimbs ((**E**), Brown–Forsythe and Welch ANOVA and Dunnett’s T3 multiple comparison test; (**F**), Kruskal–Wallis ANOVA and Dunn’s multiple comparison test; *: *p* < 0.05, **: *p* < 0.01, ***: *p* < 0.001, ****: *p* < 0.0001, *n* = 6–9 mice/group). HL-ave, average toe spread of left hindlimb.

To assess whether inhibition of CL alteration with SS-31 promotes functional recovery, a series of behavioral tests were conducted on consecutive days following SCI to evaluate motor and sensorimotor functions, including the Basso Mouse Scale (BMS), the grid walk assay (GW), the Rotorod test (RR), and TreadScan. BMS test was performed at 3 days post-injury (dpi) and weekly thereafter for 6 weeks. No significant difference in BMS scores was observed between the injured groups at 3 dpi. However, SS-31 treatment significantly improved BMS scores from 1 week up to 6 weeks post-injury in a dose-dependent manner, although the improvement in the 5 mg SS-31 group did not reach statistical significance (Figure 4A). A similar trend was observed in the effects of SS-31 treatment on Rotorod at 3 and 5 weeks post-SCI, although the improvement reached statistical significance only at 5 weeks post-SCI in the 10 mg SS-31 group (Figure 4B). There were statistically significant effects of SS-31 treatment for grid walking at 4 and 6 weeks post-SCI (Figure 4C). The quantification of average toe spread lengths using TreadScan analysis is illustrated in Figure 4D, which shows a frame of the TreadScan video after DeepLabCut (DLC) labeling. White double arrows indicate the labeled digits being compared. Quantifications of average lengths are shown in Figure 4E,F for the left and right hind paws, respectively. TreadScan analysis showed that SS-31 treatment significantly improved left hindlimb toe spreads at 7 weeks after SCI (Figure 4E), while right hindlimb toe spreads (Figure 4F) did not reach statistical significance.

### 2.5. SS-31 Did Not Reduce Tissue Damage After SCI

**Figure 5:**
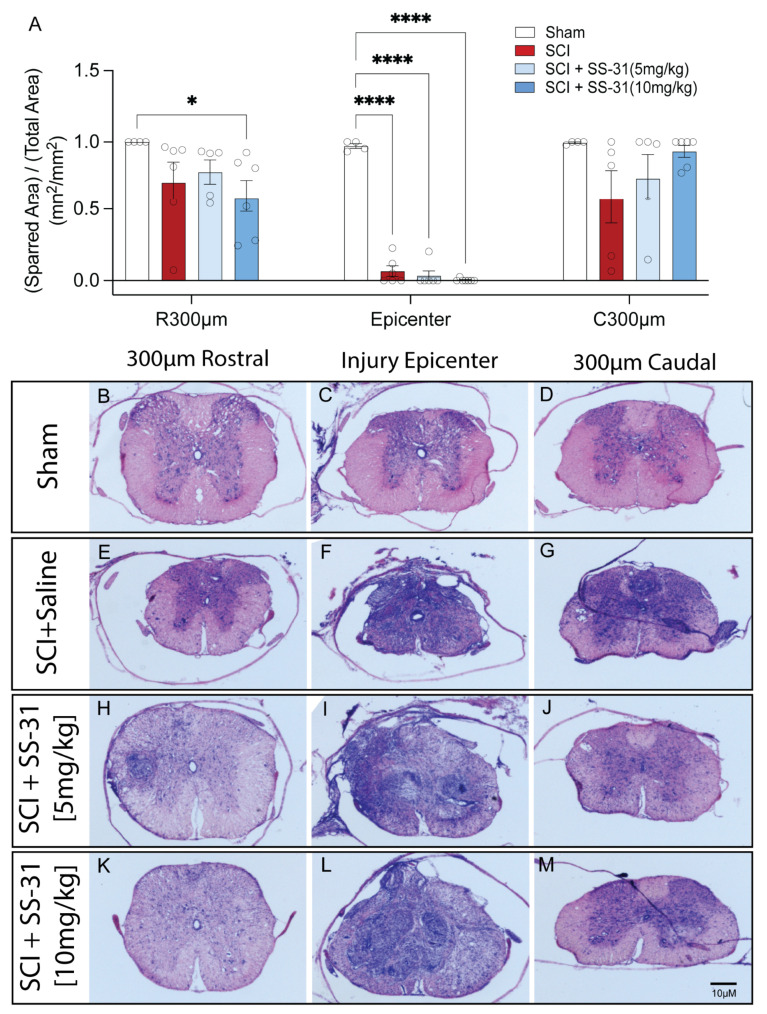
**Effect of SS-31 on tissue damage after spinal cord injury (SCI) in adult mice.** (**A**) Bar graph shows percentage of spared tissue area from 300 μm rostral to the epicenter (R300 μm) to 300 μm caudal to the epicenter (C300 μm) *: *p* < 0.05; ****: *p* < 0.0001 (Two-way ANOVA test’s treatment-factor and histology-level-factor *p*-values < 0.0001, interaction-factor *p*-value = 0.0010, *n* = 4–7/group). (**B**–**M**) Representative sections of the lesion at rostral, epicenter, and caudal levels stained with cresyl violet and eosin. Bar = 10 μm.

Because we demonstrated that administration of SS-31 significantly improved behavioral recovery after SCI, we next examined whether such treatment also would result in tissue protection in vivo. To ensure that the entire rostro-caudal expansion of the lesion was examined, a 1.2 cm-long cord segment was serially sectioned. Lesion area and spared tissue were quantified as a percentage from cresyl violet- and eosin-stained transverse sections spanning the entire lesion. There was no significant difference in the relative spared tissue at the injury epicenter of the injured mice, with consistent complete injuries that were significantly different from the sham group (Figure 5A–M). However, there was an insignificant trend toward a dose-dependent increase at 300 μm caudal to the epicenter (C300 μm) and, interestingly, there was a significant loss of spared tissue with the 10 mg/kg, but not the other two injured groups, at the level of 300 μm rostral of the epicenter (R300 μm) (Figure 5A–M).

## 3. Discussion

SCI is a devastating condition with profound physical, psychological, and socioeconomic consequences. Despite advances in understanding its pathophysiology, effective treatments remain limited [4,5]. In this study, we investigated the role of CL in secondary SCI pathology and assessed the therapeutic potential of SS-31, a CL-protective compound. Our findings demonstrate that CL levels are significantly reduced at the early stage of SCI, implicating CL alteration as a key contributor to secondary injury. Treatment with SS-31 effectively preserved CL content, mitigated mitochondrial dysfunction, and improved functional outcomes, supporting its neuroprotective potential. These results highlight the importance of CL in SCI pathology and suggest that targeting CL may offer a promising therapeutic strategy.

CL is a unique phospholipid, predominantly localized in the inner mitochondrial membrane, where it plays a critical role in maintaining mitochondrial function, including bioenergetics, membrane integrity, and apoptosis regulation [38,39,40]. Our lipidomic analysis revealed a significant reduction in CL levels 24 h after SCI, consistent with previous reports of CL peroxidation and hydrolysis in neurotrauma, including in our previous study [20] and other independent findings [23]. This loss of CL likely contributes to mitochondrial dysfunction, a hallmark of secondary injury following SCI [41,42]. SS-31, a mitochondria-targeted peptide that selectively binds to CL, effectively attenuated this CL loss in a dose-dependent manner. This protective effect aligns with SS-31’s known ability to stabilize CL and prevent its peroxidation, thereby preserving mitochondrial function [21,22,25]. Notably, SS-31 also reduced oxidized CL (OxCL), which is implicated in initiating apoptosis through cytochrome c peroxidase activity [38]. These findings suggest that CL alteration is a key mediator of mitochondrial dysfunction and neuronal death after SCI, and that SS-31’s protective effects are at least partially mediated through its interaction with CL.

In vitro experiments showed that SS-31 protects spinal cord neurons from glutamate- and rotenone-induced toxicity, two models relevant to SCI pathophysiology. Glutamate excitotoxicity and mitochondrial dysfunction are major contributors to secondary injury, leading to neuronal death and neurodegeneration [41,42,43]. SS-31 restored MMP, reduced LDH release, and decreased caspase-3/-7 activation, indicating its ability to mitigate both necrotic and apoptotic cell death. These findings are consistent with previous studies showing that SS-31 preserves mitochondrial function and reduces oxidative stress in various injury models [25,27,44,45]. Furthermore, SS-31 attenuated neurite degeneration induced by glutamate excitotoxicity, suggesting that it not only protects neurons from death but also preserves their structural integrity. This is particularly relevant in the context of SCI, where axonal damage and loss of connectivity are major contributors to functional deficits. The observed glutamate-induced cell death (~5%) differs from previous studies [46,47], which reported higher levels of cell death in spinal cord neurons exposed to glutamate. These differences may be attributed to factors such as cell type (spinal cord vs. cortical neurons), culture conditions (e.g., glutamate concentration or exposure time), and experimental timelines.

While our experiments were designed to evaluate SS-31’s effects on discrete pathways (glutamate-driven excitotoxicity and rotenone-induced mitochondrial dysfunction), we recognize that these mechanisms are interrelated—for example, excitotoxicity can exacerbate mitochondrial damage, and mitochondrial dysfunction may amplify caspase activation. Further studies directly comparing these pathways in the same model could provide additional insights into SS-31’s pleiotropic protective mechanisms.

Behavioral assessments revealed that SS-31 treatment significantly improved locomotor recovery after SCI in adult mice, as measured by the BMS, grid walk, Rotorod, and TreadScan analyses. These improvements were dose-dependent, with the 10 mg/kg dose showing the most consistent benefits. These findings are consistent with our previous report showing that inhibition of CL alteration with XJB-5-131 improves behavioral recovery after SCI in adult rats [20]. Notably, SS-31 enhanced fine motor coordination, as evidenced by improved toe spread measurements in the TreadScan analysis. These findings suggest that SS-31 not only mitigates acute mitochondrial dysfunction but also promotes long-term functional recovery, possibly by preserving neuronal connectivity and reducing secondary degeneration.

However, despite these functional improvements, SS-31 did not significantly reduce tissue damage at the injury epicenter. This discrepancy between functional recovery and tissue sparing is not uncommon in SCI research and may reflect the complexity of SCI pathophysiology [48,49]. For example, chondroitinase ABC improves locomotor recovery by promoting axonal plasticity and circuit reorganization, independent of tissue sparing [50,51]. While SS-31 effectively preserved mitochondrial function and reduced neuronal death, it may not sufficiently address other mechanisms of tissue damage, such as inflammation, glial scarring, or vascular disruption. The mitochondrial-targeted mechanism of SS-31 likely preserved neuronal survival and synaptic connectivity in surviving tissue, enabling functional recovery through neural adaptation rather than structural repair [48]. Alternatively, the lack of tissue sparing may be due to the timing or duration of SS-31 administration, as the acute phase of SCI involves multiple overlapping pathways of injury. Future studies could explore optimizing the dosing regimen or combining SS-31 with other therapies targeting complementary pathways to enhance tissue protection.

The regional differences observed at R300 μm and C300 μm may reflect variability in secondary injury responses rather than a direct detrimental effect of SS-31. A recent study reported that rostro-caudal differences in energy metabolism contribute to differential degeneration following SCI, which may explain our findings [52]. Additionally, the significant reduction at R300 μm in the 10 mg/kg group, but not the others, suggests a nonlinear dose response, which could be influenced by multiple factors, such as inflammation, gliosis, or vascular dynamics. These findings underscore the need for further investigation to clarify the underlying mechanisms and refine the therapeutic potential of SS-31 in SCI.

Several questions remain to be addressed. First, the precise mechanisms by which CL alteration contributes to mitochondrial dysfunction and neuronal death in SCI need further elucidation. While our study focused on CL peroxidation and apoptosis, other mechanisms, such as impaired mitophagy or altered lipid signaling, may also play a role. Second, the long-term effects of SS-31 on neuronal survival, axonal regeneration, and functional recovery warrant further investigation. Third, the lack of tissue sparing with SS-31 suggests that SS-31 may be most effective as part of a combinatorial approach, potentially synergizing with anti-inflammatory, neuroprotective, or regenerative therapies.

## 4. Materials and Methods

### 4.1. Reagents

All the chemicals used in this study were from Sigma (St Louis, MO, USA) except for those specifically indicated.

### 4.2. Animals

Female C57BL/6 mice (8–12 weeks, 18–24 g) were purchased from Jackson Laboratories (Bar Harbor, ME, USA). The animals were maintained on a 12/12- h light/dark cycle with food and water freely available. All surgical interventions, treatments, and postoperative animal care were performed in accordance with the Guide for the Care and Use of Laboratory Animals (National Research Council) and the Guidelines of the Institutional Animal Care and Use Committee of the Indiana University School of Medicine.

### 4.3. Spinal Cord Neuronal Culture, Cell Treatment and Viability Assessment

Primary spinal cord neurons were obtained from embryonic (E) day 15 Sprague–Dawley rat spinal cords by gentle trituration, according to our previously described protocol [9,10,20,53]. Under this culture condition, a purity greater than 85% spinal cord neuronal population was obtained at the seventh day in vitro. In this experiment, cultured spinal cord neurons were exposed to rotenone (125 nM) or glutamate (100 μM) either in the absence or presence of the designated concentrations of SS-31 for 24 h. SS-31 was added 30 min before rotenone or at the same time as glutamate administration. The cultures were maintained for an additional 24 h for MTT assay using a CellTiter 96^®^ Non-Radioactive Cell Proliferation Assay kit (Promega Corporation, Madison, WI, USA) or the culture medium was removed for LDH release assay using a CytoTox 96 Non-Radioactive Cytotoxicity Assay kit (Promega) using a microplate luminescent reader (ELx800, BioTek/Agilent, VT, USA). Real-time neurite analysis was performed using the IncuCyte ZOOM system (model-40664, ESSEN, Bioscience, Ann Arbor, MI, USA), using the Neurotrack analysis program (v.2015A1.1) (Nikon 20× objective, segmentation mode (brightness), segmentation adjustment (0.5), filtering (best), neurite sensitivity (0.6), neurite width (1 μm), min cell width cleanup (7.0 μm)). In a subset of cultures, spinal cord neurons were used for mitochondrial potential or apoptotic analysis. The larger sample size (n) for glutamate experiments was predetermined based on pilot data showing consistent low-magnitude cell death (~5%). Power analysis (α = 0.05, power = 0.8) indicated ≥15 replicates were required to reliably detect this small effect size (d = 0.3). All data represent results from three independent experiments, with each culture derived from a separate embryo/litter. “n” refers to the number of independent biological replicates, with each replicate corresponding to a separate cell culture preparation derived from different embryos/litters.

### 4.4. Mitochondrial Membrane Potential (MMP, Δψm) Assay

Mitochondrial membrane potential was assessed using the JC-1 Mitochondrial Membrane Potential Assay kit (Cayman Chemical Company, Ann Arbor, MI, USA) according to the manufacturer’s instructions and our previous publication [20]. Briefly, the JC-1 Assay Buffer was prepared and the JC-1 Stain Solution Concentrate was diluted to 1:100 in NB medium, which was mixed and further diluted 1:4 via administration as 50 μL into each well containing 150 μL of culture medium, with injury/treatment medium in the 24-well plate seeded at a density of 2.5 × 10^5^ neurons/well and incubated for 15 min at 37 °C in a CO_2_ incubator. After incubation, the cells were washed 2 times with JC-1 Assay Buffer. After washing, 200 μL of the Assay Buffer was added to each well and the cells were analyzed immediately using an the IncuCyte ZOOM system (model-40664, ESSEN, Bioscience), using the red and green image channels and the Top-Hat Analysis program (v.2015A1.1) (green acquisition time (400 ms), red acquisition time (800 ms), dual color model 4459 Nikon 20X objective, segmentation adjustment (1), cleanup hole fill (5), green and red analysis parameters (Top-Hat, 50 μm radius, (0.3) threshold, (0) edge sensitivity, 2% of red removed from green with spectral unmixing, per manufacturer’s specifications [54]). Green or red fluorescence units (GFU or RFU) were normalized to measured cell confluence for integrated intensity fluorescence units (GFU*μm^2^/image or RFU*μm^2^/image).

### 4.5. Apoptotic Analysis

Neuronal apoptosis was assessed by examining activated caspase-3/7 using the Caspase-Glo^®^ 3/7 Assay System (Promega) according to the manufacturer’s instructions modified for 24-well plates. Briefly, at 3 h post-treatment, cultured spinal cord neurons were incubated with a 100X stock solution of Caspase-Glo^®^ 3/7 Reagent at a 1:100 dilution for 1 h, after which, at 4 h (a time point previously reported for apoptosis following SCI [36,55]), images were automatically captured and analyzed fluorescence proportional to activated caspase-3/7 relative to cell confluence using the IncuCyte ZOOM system (ESSEN, Bioscience) photographing in phase and green image channels followed by Top-Hat Analysis program (green acquisition time (450 ms), dual color model 4459 Nikon 20X objective, segmentation adjustment (1), cleanup hole fill (5), green analysis parameters (Top-Hat, 10 μm radius, (0.3) threshold, (0) edge sensitivity, per manufacturer specifications [54]). Green fluorescence units (GFU) were normalized to measured cell confluency for integrated intensity fluorescence units (GFU*μm^2^/image).

### 4.6. Spinal Cord Injury and Treatment

Spinal cord contusion injury was produced using an Infinity Horizon (IH) Impactor (version R5.0.4, Precision Systems and Instrumentation, Lexington, KY, USA) according to a previously published method [10,20]. Briefly, mice were anesthetized with an intraperitoneal (i.p.) injection of an anesthetic cocktail, including ketamine (100 mg/kg)/xylazine (4 mg/kg)/acepromazine (2 mg/kg), and a laminectomy was performed at the T9-T10 level. After the vertebral column was stabilized by a vertebral stabilizer, the exposed dorsal surface of the cord was subjected to an impact of 60 kDyne force. For any injury where the impactor reported a sharp peak of the impactor, it was presumed that the impactor had hit bone and a failed SCI was induced, so those mice were removed from the study. For the sham-injury controls, the animals underwent the laminectomy without the impact. After SCI impact, immediate spinal cord bruising was visually confirmed and the layers of muscle and skin at the site of exposure were sutured. Then animals were placed in the cage on a heating pad until full recovery from anesthesia. Manual bladder expression was carried out at least twice daily until reflex bladder emptying was reestablished.

For acute lipidomic analysis, mice were sacrificed at 24 h after SCI, having only received a single dose of SS-31 immediately (0, 5, or 10 mg/kg in saline, i.p.) after injury. The spinal cord segments (10 mm) containing the injury epicenter were removed for lipidomics. The 24-h time point was chosen because cardiolipin alteration is an early event following SCI [20]. For subacute and intermediate locomotor and histological analysis, the mice were received daily doses of SS-31 (0, 5, or 10 mg/kg in saline, i.p.) for the first 6 days post-injury (7 total injections). At 8 weeks post-injury, mice were sacrificed, and their tissues were processed for histological analysis.

### 4.7. Lipidomics

Lipidomics analysis was performed following our previously described protocol [20]. Mice were anesthetized with an intraperitoneal (i.p.) injection of an anesthetic cocktail containing ketamine (100 mg/kg)/xylazine (4 mg/kg)/acepromazine (2 mg/kg). After sacrifice, a 10 mm segment of the spinal cord containing the injury was rapidly dissected. Each segment was immediately flash-frozen in liquid nitrogen and stored at −80 °C until shipped overnight on dry ice to the Lipidomics Service at Barshop Institute (UT Health, San Antonio, TX, USA). Lipid extraction and lipidomics analysis were performed blindly by the Lipidomics Service using mass spectrometry based lipidomics.

### 4.8. Behavioral Assessments

The BMS locomotor test was performed, starting 3 days post-SCI, weekly up to 6 weeks post-SCI by two observers lacking knowledge of the experimental groups, according to a previously published method [10,56]. BMS is a 9-point scale, in which 0 signifies total paralysis and 9 reflects normal locomotion, with scores accounting for grades of SCI-affected kinematics (stepping, ankle movement, coordination, trunk stability, tail elevation and paw placement) [56].

The Rotorod (RR) test was used to assess sensorimotor deficits, following our previously described method with minor modifications [57]. Latency for balancing on the rotating rod were evaluated at 3 and 5 weeks post-injury. During the test, the mice were placed on the stationary rod for a 10 s to acclimate before the test, then the rod was made to rotate at a rate of that started at 1 rotation per minute (rpm) and that rate of rotation accelerated to a speed of 30 rpm over the course of 90 s, then that maximum rotational speed held until for 120 s max time. Trials ended when the animal either fell off the rod or clung to the rod as it made one complete rotation. Following injury, animals were given three acceleration trials each. Individual scores from these trials were averaged and evaluated.

Grid walking (GW) was used also to assess hindlimb locomotor deficits [20,53,58,59], using a modified grid (1 cm^2^) for mice. Foot falls were evaluated at 4 and 6 weeks post-injury. During the test, the mice were allowed to walk on an elevated metal rectangle grid (40 cm above a tabletop). Total hindlimb footfalls were counted by two observers, unaware of the experimental groups during each trial. For testing, each animal was placed on the grid and allowed to perform active grid walking task for a period of 3 min. During this time period, the number of foot falls (fall of the hindlimb, including at least the ankle joint, through the grid surface) was determined individually for each hindlimb.

Gait analysis using TreadScan (version 4.00, CleverSys Inc., Reston, VA, USA) allowed highly sensitive, noninvasive evaluation of extremities. TreadScan utilizes a transparent treadmill belt and a high-speed (100 frames/s) camera to capture the videos of animals gait characteristics [60]. At 7 weeks post-injury, all mice were made to walk on the motor-driven treadmill belt at a speed of 7 cm/s for 20 s, as described previously [60,61]. A highspeed digital video camera recorded all the movements. The digital data locomotion after SCI were analyzed by Pose-estimation machine learning software, DeepLabCut (DLC, GUI v2.2.3).

### 4.9. Histological Assessments

Following behavioral analysis, at 8 weeks post-injury, all of the mice were sacrificed via pneumothorax perfusion, followed by spinal cord dissection for histology analysis. Spinal cord segments (1.2 cm, centered at the injury epicenter) were isolated from each animal, embedded, and sectioned into 25 μm-thick serial sections (250 μm apart and spanning the entire rostro caudal extent of the lesion). One set of the sections was stained with cresyl violet–eosin. The lesion and spared area of the injured cord were visualized, outlined, and quantified using an Olympus BX60 microscope equipped with a Neurolucida system (MicroBrightField, Colchester, VT, USA).

### 4.10. Statistical Analysis

All statistical analyses were performed using GraphPad Prism software (version 10.0.2, La Jolla, CA, USA). All data are presented as mean ± s.e.m. For single time-point data, normality and unequal variance were measured using the Shapiro–Wilks and Levene or Brown–Forsythe tests. Accordingly, the (non-normal), (normal but unequally variant), and (normal and equally variant) datasets were analyzed respectively with the (Kruskal–Wallis ANOVA, followed by Dunn’s multiple comparison test), (Welch’s ANOVA followed by Dunnett’s T3 test), and (one-way ANOVA, followed by Tukey’s post hoc test). For grouped data, the mixed-effects two-way ANOVA was used followed by Tukey’s post hoc test. A *p* value of < 0.05 was considered statistically significant.

## 5. Conclusions

In conclusion, this study demonstrates that CL alteration is a key contributor to mitochondrial dysfunction and neuronal death after SCI, and that SS-31 effectively attenuates these changes, leading to improved functional recovery. While SS-31 did not significantly reduce tissue damage, its ability to preserve mitochondrial function and neuronal integrity highlights its potential as a therapeutic agent for SCI. However, some limitations remain. First, the precise mechanisms by which CL alteration contributes to mitochondrial dysfunction and neuronal death in SCI need further elucidation. While our study focused on CL peroxidation and apoptosis, other mechanisms, such as impaired mitophagy or altered lipid signaling, may also play a role. Additionally, SS-31’s inability to reduce tissue damage suggests its benefits are primarily functional, indicating a need for complementary interventions to enhance tissue sparing. Second, the study did not assess long-term neurophysiological changes, such as synaptic plasticity or corticospinal tract integrity, which are critical for sustained recovery. Future studies using electrophysiological or tract-tracing techniques could provide further insights. Third, the use of a single time point for lipidomic and biochemical analyses limits our understanding of the dynamic evolution of mitochondrial dysfunction post-SCI. A time-course study could better define CL alterations and identify the optimal therapeutic window. Finally, the lack of tissue sparing with SS-31 suggests that it may be most effective as part of a combinatorial approach, potentially synergizing with anti-inflammatory, neuroprotective, or regenerative therapies to enhance overall recovery. In summary, these findings advance our understanding of SCI pathophysiology and provide a foundation for developing novel, mitochondria-targeted treatments for this devastating condition. Future studies should focus on optimizing SS-31 treatment protocols, clarifying molecular pathways, and exploring combination with other therapies to maximize its neuroprotective and regenerative effects.

## Data Availability

Data are contained within the article.

## References

[1] Sinescu C., Popa F., Grigorean V., Onose G., Sandu A., Popescu M., Burnei G., Strambu V., Popa C. (2010). Molecular basis of vascular events following spinal cord injury. J. Med. Life.

[2] GBD 2016 Traumatic Brain Injury and Spinal Cord Injury Collaborators (2019). Global, regional, and national burden of traumatic brain injury and spinal cord injury, 1990–2016: A systematic analysis for the Global Burden of Disease Study 2016. Lancet Neurol..

[3] Fehlings M.G., Singh A., Tetreault L., Kalsi-Ryan S., Nouri A. (2014). Global prevalence and incidence of traumatic spinal cord injury. Clin. Epidemiol..

[4] Rabchevsky A.G., Patel S.P., Springer J.E. (2011). Pharmacological interventions for spinal cord injury: Where do we stand? How might we step forward?. Pharmacol. Ther..

[5] Liu N.K., Xu X.M. (2012). Neuroprotection and its molecular mechanism following spinal cord injury. Neural Reg. Res..

[6] Liu N.-K., Xu X.-M. (2010). Phospholipase A2 and its Molecular Mechanism after Spinal Cord Injury. Mol. Neurobiol..

[7] Farooqui A.A., Horrocks L.A. (2006). Phospholipase A2-generated lipid mediators in the brain: The good, the bad, and the ugly. Neuroscientist.

[8] Sullivan P.G., Rabchevsky A.G., Keller J.N., Lovell M., Sodhi A., Hart R.P., Scheff S.W. (2004). Intrinsic differences in brain and spinal cord mitochondria: Implication for therapeutic interventions. J. Comp. Neurol..

[9] Liu N.-K., Zhang Y.P., Titsworth W.L., Jiang X., Han S., Lu P.-H., Shields C.B., Xu X.-M. (2006). A novel role of phospholipase A2in mediating spinal cord secondary injury. Ann. Neurol..

[10] Liu N., Deng L., Zhang Y.P., Lu Q., Wang X., Hu J., Oakes E., Bonventre J.V., Shields C.B., Xu X. (2014). Cytosolic phospholipase A_2_ protein as a novel therapeutic target for spinal cord injury. Ann. Neurol..

[11] Liu N.-K., Titsworth W., Xu X.-M., Lajtha A., Banik N., Ray S.K. (2009). Phospholipase A2 in CNS disorders: Implication on traumatic spinal cord and brain injuries. Handbook of Neurochemistry and Molecular Neurobiology.

[12] Farooqui A.A., Ong W.-Y., Horrocks L.A. (2004). Biochemical Aspects of Neurodegeneration in Human Brain: Involvement of Neural Membrane Phospholipids and Phospholipases A2. Neurochem. Res..

[13] Christie W.W., Han X. (2012). Chapter 1—Lipids: Their structures and occurrence. Lipid Analysis.

[14] Hall E.D., Braughler J.M. (1986). Role of Lipid Peroxidation in Post-Traumatic Spinal Cord Degeneration: A Review. Central Nerv. Syst. Trauma.

[15] Braughler J.M., Hall E.D. (1992). Involvement of lipid peroxidation in CNS injury. J. Neurotrauma.

[16] Shamas-Din A., Bindner S., Chi X., Leber B., Andrews D.W., Fradin C. (2015). Distinct lipid effects on tBid and Bim activation of membrane permeabilization by pro-apoptotic Bax. Biochem. J..

[17] Paradies G., Paradies V., Ruggiero F.M., Petrosillo G. (2019). Role of Cardiolipin in Mitochondrial Function and Dynamics in Health and Disease: Molecular and Pharmacological Aspects. Cells.

[18] Schlame M., Rua D., Greenberg M.L. (2000). The biosynthesis and functional role of cardiolipin. Prog. Lipid Res..

[19] Mohammadyani D., Yanamala N., Samhan-Arias A.K., Kapralov A.A., Stepanov G., Nuar N., Planas-Iglesias J., Sanghera N., Kagan V.E., Klein-Seetharaman J. (2018). Structural characterization of cardiolipin-driven activation of cytochrome c into a peroxidase and membrane perturbation. Biochim. Biophys. Acta (BBA)—Biomembr..

[20] Liu N.-K., Deng L.-X., Wang M., Lu Q.-B., Wang C., Wu X., Wu W., Wang Y., Qu W., Han Q. (2022). Restoring mitochondrial cardiolipin homeostasis reduces cell death and promotes recovery after spinal cord injury. Cell Death Dis..

[21] Zhu Y., Wang H., Fang J., Dai W., Zhou J., Wang X., Zhou M. (2018). SS-31 Provides Neuroprotection by Reversing Mitochondrial Dysfunction after Traumatic Brain Injury. Oxidative Med. Cell. Longev..

[22] Zhu L.-L., Li M.-Q., He F., Zhou S.-B., Jiang W. (2017). Mitochondria Targeted Peptide Attenuates Mitochondrial Dysfunction, Controls Inflammation and Protects Against Spinal Cord Injury-Induced Lung Injury. Cell. Physiol. Biochem..

[23] Ji J., Kline A.E., Amoscato A., Samhan-Arias A.K., Sparvero L.J., Tyurin V.A., Tyurina Y.Y., Fink B., Manole M.D., Puccio A.M. (2012). Lipidomics identifies cardiolipin oxidation as a mitochondrial target for redox therapy of brain injury. Nat. Neurosci..

[24] Birk A.V., Chao W.M., Bracken C., Warren J.D., Szeto H.H. (2014). Targeting mitochondrial cardiolipin and the cytochrome *c*/cardiolipin complex to promote electron transport and optimize mitochondrial ATP synthesis. Br. J. Pharmacol..

[25] Szeto H.H. (2014). First-in-class cardiolipin-protective compound as a therapeutic agent to restore mitochondrial bioenergetics. Br. J. Pharmacol..

[26] Zhao K., Zhao G.-M., Wu D., Soong Y., Birk A.V., Schiller P.W., Szeto H.H. (2004). Cell-permeable Peptide Antioxidants Targeted to Inner Mitochondrial Membrane inhibit Mitochondrial Swelling, Oxidative Cell Death, and Reperfusion Injury. J. Biol. Chem..

[27] Birk A.V., Liu S., Soong Y., Mills W., Singh P., Warren J.D., Seshan S.V., Pardee J.D., Szeto H.H. (2013). The Mitochondrial-Targeted Compound SS-31 Re-Energizes Ischemic Mitochondria by Interacting with Cardiolipin. J. Am. Soc. Nephrol..

[28] Graham Z.A., DeBerry J.J., Cardozo C.P., Bamman M.M. (2021). A 50 kdyne contusion spinal cord injury with or without the drug SS-31 was not associated with major changes in muscle mass or gene expression 14 d after injury in young male mice. Physiol. Rep..

[29] Graham Z.A., DeBerry J.J., Cardozo C.P., Bamman M.M. (2022). SS-31 does not prevent or reduce muscle atrophy 7 days after a 65 kdyne contusion spinal cord injury in young male mice. Physiol. Rep..

[30] Zhang H., Chen Y., Li F., Wu C., Cai W., Ye H., Su H., He M., Yang L., Wang X. (2023). Elamipretide alleviates pyroptosis in traumatically injured spinal cord by inhibiting cPLA2-induced lysosomal membrane permeabilization. J. Neuroinflamm..

[31] Jiang W., He F., Ding G., Wu J. (2023). Elamipretide reduces pyroptosis and improves functional recovery after spinal cord injury. CNS Neurosci. Ther..

[32] Chu T.C., Ji J., Dagda R.K., Jiang J.F., Tyurina Y.Y., Kapralov A.A., Tyurin V.A., Yanamala N., Shrivastava I.H., Mohammadyani D. (2013). Cardiolipin externalization to the outer mitochondrial membrane acts as an elimination signal for mitophagy in neuronal cells. Nat. Cell Biol..

[33] Guo W.-X., Pye Q.N., Williamson K.S., Stewart C.A., Hensley K.L., Kotake Y., Floyd R.A., Broyles R.H. (2005). Mitochondrial dysfunction in choline deficiency-induced apoptosis in cultured rat hepatocytes. Free Radic. Biol. Med..

[34] Salido M., Gonzalez J.L., Vilches J. (2007). Loss of mitochondrial membrane potential is inhibited by bombesin in etoposide-induced apoptosis in PC-3 prostate carcinoma cells. Mol. Cancer Ther..

[35] Salvioli S., Ardizzoni A., Franceschi C., Cossarizza A. (1997). JC-1, but not DiOC6(3) or rhodamine 123, is a reliable fluorescent probe to assess delta psi changes in intact cells: Implications for studies on mitochondrial functionality during apoptosis. FEBS Lett..

[36] Wingrave J.M., Schaecher K.E., Sribnick E.A., Wilford G.G., Ray S.K., Hazen-Martin D.J., Hogan E.L., Banik N.L. (2003). Early induction of secondary injury factors causing activation of calpain and mitochondria-mediated neuronal apoptosis following spinal cord injury in rats. J. Neurosci. Res..

[37] Bayir H., Tyurin V.A., Tyurina Y.Y., Viner R., Ritov V., Amoscato A.A., Zhao Q., Zhang X.J., Janesko-Feldman K.L., Alexander H. (2007). Selective early cardiolipin peroxidation after traumatic brain injury: An oxidative lipidomics analysis. Ann. Neurol..

[38] Kagan V.E., Tyurin V.A., Jiang J., Tyurina Y.Y., Ritov V.B., Amoscato A.A., Osipov A.N., Belikova N.A., Kapralov A.A., Kini V. (2005). Cytochrome c acts as a cardiolipin oxygenase required for release of proapoptotic factors. Nat. Chem. Biol..

[39] Chicco A.J., Sparagna G.C. (2007). Role of cardiolipin alterations in mitochondrial dysfunction and disease. Am. J. Physiol. Cell Physiol..

[40] Gonzalvez F., Gottlieb E. (2007). Cardiolipin: Setting the beat of apoptosis. Apoptosis.

[41] Liu C., Liu Y., Ma B., Zhou M., Zhao X., Fu X., Kan S., Hu W., Zhu R. (2022). Mitochondrial regulatory mechanisms in spinal cord injury: A narrative review. Medicine.

[42] Sullivan P.G., Krishnamurthy S., Patel S.P., Pandya J.D., Rabchevsky A.G. (2007). Temporal Characterization of Mitochondrial Bioenergetics after Spinal Cord Injury. J. Neurotrauma.

[43] Park E., Velumian A.A., Fehlings M.G. (2004). The Role of Excitotoxicity in Secondary Mechanisms of Spinal Cord Injury: A Review with an Emphasis on the Implications for White Matter Degeneration. J. Neurotrauma.

[44] Szeto H.H., Birk A.V. (2014). Serendipity and the Discovery of Novel Compounds That Restore Mitochondrial Plasticity. Clin. Pharmacol. Ther..

[45] Siegel M.P., Kruse S.E., Percival J.M., Goh J., White C.C., Hopkins H.C., Kavanagh T.J., Szeto H.H., Rabinovitch P.S., Marcinek D.J. (2013). Mitochondrial-targeted peptide rapidly improves mitochondrial energetics and skeletal muscle performance in aged mice. Aging Cell.

[46] Regan R., Choi D. (1991). Glutamate neurotoxicity in spinal cord cell culture. Neuroscience.

[47] Zhou L., Li F., Xu H.-B., Luo C.-X., Wu H.-Y., Zhu M.-M., Lu W., Ji X., Zhou Q.-G., Zhu D.-Y. (2010). Treatment of cerebral ischemia by disrupting ischemia-induced interaction of nNOS with PSD-95. Nat. Med..

[48] Bradbury E.J., Moon L.D.F., Popat R.J., King V.R., Bennett G.S., Patel P.N., Fawcett J.W., McMahon S.B. (2002). Chondroitinase ABC promotes functional recovery after spinal cord injury. Nature.

[49] García-Alías G., Barkhuysen S., Buckle M., Fawcett J.W. (2009). Chondroitinase ABC treatment opens a window of opportunity for task-specific rehabilitation. Nat. Neurosci..

[50] Fouad K., Schnell L., Bunge M.B., Schwab M.E., Liebscher T., Pearse D.D. (2005). Combining Schwann Cell Bridges and Olfactory-Ensheathing Glia Grafts with Chondroitinase Promotes Locomotor Recovery after Complete Transection of the Spinal Cord. J. Neurosci..

[51] Rosenzweig E.S., Courtine G., Jindrich D.L., Brock J.H., Ferguson A.R., Strand S.C., Nout Y.S., Roy R.R., Miller D.M., Beattie M.S. (2010). Extensive spontaneous plasticity of corticospinal projections after primate spinal cord injury. Nat. Neurosci..

[52] Ohnishi Y., Yamamoto M., Sugiura Y., Setoyama D., Kishima H. (2021). Rostro-caudal different energy metabolism leading to differences in degeneration in spinal cord injury. Brain Commun..

[53] Liu N.-K., Zhang Y.P., Han S., Pei J., Xu L.Y., Lu P.-H., Shields C.B., Xu X.-M. (2007). Annexin A1 Reduces Inflammatory Reaction and Tissue Damage Through Inhibition of Phospholipase A2 Activation in Adult Rats Following Spinal Cord Injury. J. Neuropathol. Exp. Neurol..

[54] Essen Bioscience Incucyte Live-Cell Analysis System—Technical Note. https://www.sartorius.com/download/1163270/incucyte-basic-analysis-software-guidelines-8000-0522-d00-data.pdf.

[55] Lou J., Lenke L.G., Ludwig F.J., O’Brien M.F. (1998). Apoptosis as a mechanism of neuronal cell death following acute experimental spinal cord injury. Spinal Cord.

[56] Basso D.M., Fisher L.C., Anderson A.J., Jakeman L.B., Mctigue D.M., Popovich P.G. (2006). Basso Mouse Scale for Locomotion Detects Differences in Recovery after Spinal Cord Injury in Five Common Mouse Strains. J. Neurotrauma.

[57] Liu N.-K., Zhang Y.-P., O’Connor J., Gianaris A., Oakes E., Lu Q.-B., Verhovshek T., Walker C.L., Shields C.B., Xu X.-M. (2013). A bilateral head injury that shows graded brain damage and behavioral deficits in adultmice. Brain Res..

[58] Schucht P., Raineteau O., Schwab M.E., Fouad K. (2002). Anatomical correlates of locomotor recovery following dorsal and ventral lesions of the rat spinal cord. Exp. Neurol..

[59] Metz G.A., Merkler D., Dietz V., Schwab M.E., Fouad K. (2000). Efficient testing of motor function in spinal cord injured rats. Brain Res..

[60] Hamers F.P., Lankhorst A.J., van Laar T.J., Veldhuis W.B., Gispen W.H. (2001). Automated Quantitative Gait Analysis During Overground Locomotion in the Rat: Its Application to Spinal Cord Contusion and Transection Injuries. J. Neurotrauma.

[61] Beare J.E., Morehouse J.R., DeVries W.H., Enzmann G.U., Burke D.A., Magnuson D.S., Whittemore S.R. (2009). Gait Analysis in Normal and Spinal Contused Mice Using the TreadScan System. J. Neurotrauma.

